# Bronchodilatation increases number of particles in exhaled air in subjects with asthma

**DOI:** 10.1186/2045-7022-3-S1-P12

**Published:** 2013-05-03

**Authors:** Anna-Carin Olin, Emilia Viklund, Per Larsson, Ann-Charlotte Almstrand, Anna Bredberg, Ekaterina Mirgorodskaya, Björn Bake

**Affiliations:** 1 Gothenburg University, Occupational and Environmental Medicine, Sweden; 2 Gothenburg University, Dept of Respiratory Medicine and Allergology, Sweden

## Background

Particles in exhaled air (PEx) are derived from the small airways and are formed during airway closure and re-opening. They mainly contain surfactant; both phospholipid and protein composition in PEx resemble that of BAL. Measurements of surfactant protein A in PEx from 100 l exhaled air were shown to be highly reproducible, making the PEx a promising tool in the monitoring of asthma. The number of exhaled particles varies substantially, mainly among subjects, but also within subjects. To enable a correct interpretation of the results using PEx it is crucial to examine how airway constriction affects the number of exhaled particles.

## Aim

To examine the effect of bronchodilatation on exhaled PEx concentration.

## Method

16 subjects with pollen-asthma and 14 healthy non-atopic subjects (all non-smokers) were examined before and after bronchodilation during the pollen season and outside the pollen-season. PEx, spirometry, blood-samples and answers to a questionnaire were obtained. The subjects performed a breathing maneuvers allowing for airway closure and re-opening and PEx concentrations in about 60 l of exhaled air were measured with an in-house developed instrument based on particle impaction.

## Results

PEx concentrations were not significantly different between asthmatics and controls but asthmatics showed lower PEx concentrations during pollen season compared to outside pollen season (3.46 v s 4.32 p=0.01) whereas controls showed non-significant differences between seasons (6.86 v s 4.54 p=0.15). PEx concentrations increased after bronchodilatation in asthmatics (median 4.05*103 to 4.92*103, p=0.02), but not in controls (median 4.47* 103 v s 4.50*103 p=0.12). The change in PEx concentration (%) was associated with the change in FVC (%) (rp= 0.51, p=0.001) and FEV1 (rp= 0.46, p=0.003) among subjects with asthma whereas there were no significant correlations among controls.

## Conclusion

In the present study the subjects had mild symptoms and rather low reversibility also during pollen-season. Nevertheless, PEx concentrations were apparently influenced by bronchomotor tone and increased after bronchodilatation, presumably reflecting increased airway opening following bronchodilatation in asthmatics with ongoing airway inflammation.

